# Exercise during early, but not late abstinence, attenuates subsequent relapse vulnerability in a rat model

**DOI:** 10.1038/tp.2016.58

**Published:** 2016-04-26

**Authors:** R M Beiter, A B Peterson, J Abel, W J Lynch

**Affiliations:** 1 Department of Psychiatry and Neurobehavioral Sciences, University of Virginia, Charlottesville, VA, USA

## Abstract

Exercise has shown promise as a nonpharmacological intervention for addiction, with evidence suggesting a potential utility for relapse prevention. In humans, exercise as an intervention is typically introduced well after the initiation of abstinence, yet neurobiological data from preclinical studies suggest that it may be more effective if initiated during early abstinence. Here, using rat models, we determined whether the beneficial effects of exercise on relapse vulnerability depends on when exercise is first initiated, during early versus late abstinence. Once rats (*n*=47) acquired cocaine self-administration, they were given 24-h access to cocaine (1.5 mg/kg per infusion) under a discrete trial procedure (four infusions per hour) for 10 days. The rats then began a 14-day abstinence period in which they had access (2 h per day) to a locked wheel throughout abstinence (sedentary) or an unlocked wheel during early (days 1–7), late (days 8–14) or throughout (days 1–14) abstinence (*n*=10–14 per group). Cocaine seeking, as assessed under an extinction/cued-induced reinstatement procedure, was examined on day 15 of abstinence. Exercise beginning during early abstinence robustly attenuated subsequent cocaine seeking, and this effect persisted even when exercise ended on the seventh day of abstinence. In contrast, exercise during late abstinence was not effective and these animals displayed high levels of cocaine seeking similar to those observed in sedentary animals. These results indicate that the timing of exercise availability differentially impacts cocaine seeking with results suggesting that exercise during early, but not late, abstinence may provide long-term protection against cocaine relapse.

## Introduction

Drug addiction is a leading cause of preventable death in the United States.^1^ Although several medications with modest efficacy have been approved for the treatment of alcohol, opioid and tobacco addiction, none have been approved for the treatment of cocaine addiction.^2, 3^ This deficit in effective treatments has led researchers to explore other methods, including exercise-based interventions. Epidemiological studies have long reported negative associations between levels of physical activity and illicit drug use.^4, 5^ There are also many anecdotal reports suggesting that people in recovery turn to exercise to help maintain abstinence. Exercise has been shown to reduce symptoms of depression and measures of anxiety, and to enhance cognition,^6, 7^ factors known to contribute to drug relapse.^8, 9^ Recent reports analyzing exercise-based programs piloted at drug abuse treatment facilities have also shown that engaging in a consistent exercise regimen reduces withdrawal symptoms, increases the likelihood of remaining abstinent and decreases craving.^10, 11, 12, 13^ In animals, several studies have shown that wheel and treadmill running reduce the reinforcing effects of cocaine, as well as cocaine seeking when the exercise option is contemporaneously available.^14^ Our recent work in males and the work of others also supports its potential utility at reducing relapse vulnerability, with results showing that wheel-running exercise during abstinence attenuates subsequent cocaine seeking.^15, 16, 17, 18^

Despite these promising results, however, there is evidence that certain exercise conditions may be either ineffective or lead to detrimental effects. For example, in humans, the long-term efficacy of exercise at reducing smoking is controversial,^19^ and there are several reports indicating that involvement in certain team sports can be associated with higher levels of drug use.^20, 21^ Although this latter finding is likely attributable in part to social factors,^22^ findings in animals have also revealed that certain exercise conditions enhance rather than decrease addiction-related behaviors^23, 24^ suggesting that biological factors may also contribute. Given that exercise is becoming more frequently considered as a potential treatment for addiction, it is critical to identify not only the conditions that produce beneficial effects, but also those that are ineffective or lead to detrimental effects.

We recently proposed that the efficacy of exercise as an intervention for addiction depends on the neurobiological mechanisms underlying the drug-motivated behavior, which varies from early to later periods of abstinence.^14^ Numerous neurobiological attributes vary concurrently with the time elapsed since the beginning of an abstinence period, including brain-derived neurotrophic factor (BDNF), dopamine, brain glucose metabolism, expression of μ-opioid receptors and brain region activation.^25, 26, 27, 28, 29, 30, 31^ In addition, cocaine seeking increases over a period of abstinence, a phenomenon known as incubation of cocaine craving.^29^ Evidence suggests that the decrease in cocaine seeking observed during early abstinence is influenced by a deficit in neurobiological processes, including BDNF, dopamine, ERK activity and various other signaling pathways.^32^ The subsequent increase in cocaine seeking is thought to be governed by a compensatory increase in these same neurobiological factors, which have been shown to exhibit time-dependent increases throughout abstinence.^28, 29, 33, 34^ These fluctuations in neurobiological systems throughout abstinence suggest that the efficacy of exercise may differ when offered immediately upon initiation of abstinence as compared with beginning during later abstinence. Specifically, exercise is known to increase neurotransmission of numerous signaling pathways including dopamine and BDNF.^35, 36^ Thus, when utilized during early, but not late abstinence, it may prevent the overcompensation of these systems that lead to the incubation of cocaine seeking following extended abstinence.^15, 16, 37^ This possibility has not yet been examined in either humans or animal models.

In this study, we compared the effects of wheel-running exercise during early (days 1–7) versus late (days 8–14) abstinence on subsequent cocaine seeking in the male rats following extended access to cocaine self-administration. We also compared the effects of early or late exercise to the effects of access to a locked or unlocked wheel throughout abstinence (days 1–14). On the basis of the neurobiological data,^29, 33, 38^ we predicted that exercise during early abstinence would cause a greater decrease in cocaine seeking as compared with exercise during late abstinence.

## Materials and methods

### Subjects

Adult male (*n*=47) Sprague Dawley rats weighing between 360 and 410 g at the beginning of the study were used as subjects. Upon arrival to the laboratory, the rats were housed individually in operant conditioning chambers (Med Associates, St. Albans, VT, USA) with *ad libitum* access to food and water. The rats remained in these chambers throughout the experiment except during the 14-day abstinence period as detailed below. Both the testing room and the individual operant chambers were maintained on a 12-h light/dark cycle (room and house lights on at 0700 h). After a 1–2-day acclimation period, the rats were pre-trained to lever press for sucrose pellets (45 mg; Noyes Company, Lancaster, NH, USA) using a brief training protocol and methods previously described;^39^ fixed-ratio 1 (FR1); 23 h per day sessions until the rats obtained 50 or more pellets for two consecutive sessions. Lever-press training was used to ensure that subsequent cocaine self-administration would be rapidly acquired. Each rat was then implanted with a jugular catheter using methods previously described.^40^ Catheter patency was tested for two consecutive days following the surgery and thereafter 3 days per week by flushing the catheter with a small amount of heparinized saline and then pulling back until blood appeared in the line. Cocaine self-administration training (as detailed below) was initiated once grooming and eating behaviors resumed (typically after 48 h of recovery). The rats were weighed three times per week and health was monitored daily. All the procedures were approved by the University of Virginia Animal Care and Use Committee and were conducted within the guidelines set by the NIH.

### Drugs

Cocaine hydrochloride was obtained from the NIDA, dissolved in sterile saline and filter-sterilized using a 0.22 micrometer millipore filter. The same milligram per milliliter concentration was maintained throughout the study with the infusion duration adjusted three times per week based on body weight (2 s per 100 g) to maintain a constant cocaine dose (1.5 mg/kg per infusion).

### Procedure

#### Cocaine self-administration

The rats were trained to self-administer cocaine (1.5 mg/kg per infusion) during daily sessions under an FR1 schedule with a maximum of 20 infusions available per day as previously described.^15^ Following acquisition (defined as two consecutive sessions wherein all 20 infusions were obtained), the rats were given 24-h access to cocaine under a discrete trial procedure. Each discrete 10-min trial began with the introduction of the left lever into the chamber, and each response on this lever during a trial produced an infusion of cocaine (1.5 mg/kg per infusion), and the illumination of a stimulus light above the lever (paired with the infusion; approximately 5 s). A discrete trial was terminated and the lever was retracted after either a response on this lever under an FR1 schedule or after 10 min had elapsed. The trials initiated every 15 min for a total of 10 days. The right inactive lever was extended into the chamber throughout the experiment, and responses on it were recorded but did not produce a consequence. The 1.5 mg/kg per infusion dose was selected for both cocaine self-administration training and extended-access self-administration as this relatively high dose produces maximal and rapid rates of acquisition, as well as high levels of intake under the discrete trial procedure.^41^ Following the last discrete trial session, two additional sessions of FR1 self-administration (maximum of 20 infusions) were run to equate all the groups on intake before abstinence and to confirm catheter patency. The rats were then randomly assigned to one of four abstinence conditions: sedentary (sedentary; *n*=11), early exercise (early; during days 1–7 of abstinence; *n*=12), late exercise (late; during days 8–14 of abstinence; *n*=14) or throughout exercise (throughout; during days 1–14; *n*=10). We have previously demonstrated that a sample size of eight is sufficient for detecting effects of exercise throughout abstinence,^15, 16, 17^ and increasing the sample size to a minimum of 10 per group here allowed for a determination of timing of exercise availability effects (based on a power level of 0.8 and significance level of 0.05). One sedentary rat had to be removed from the study before completion due to illness; the data from this animal were removed from all the statistical analyses (final, *n*=10).

#### Wheel running during abstinence

A 14-day abstinence period began following the last cocaine self-administration session during which the rats were housed individually in polycarbonate cages with a 12.5″-diameter running wheel (Med Associates) attached. This abstinence length was selected on the basis of results showing that cocaine seeking incubates over abstinence with high levels observed following 14 days of abstinence.^33^ The rats assigned to an exercise group were given access to a running wheel for 2 h per day with sessions beginning between 0900 and 1100 h. We have previously shown that this wheel-running session length (2 h per day) effectively blocks the development of the incubation effect when available throughout abstinence.^15, 16, 17^ The sessions were conducted daily during early (days 1–7), late (days 8–14) or throughout abstinence (days 1–14). The rats in the sedentary group were also given access to a wheel for 2 h per day throughout abstinence; however, the wheel was locked at all times (as a control for environmental enrichment). Although the throughout and sedentary groups had access to a wheel (either locked or unlocked) throughout the entire abstinence period while the early and late groups only had access to the wheel for 7 out of 14 abstinence days, we have previously shown that the locked-wheel condition and polycarbonate housing have the same effects on subsequent cocaine-seeking behavior.^17^ When wheel running was unavailable (that is, days 8–14 for the early group and days 1–7 for the late group), a metal partition separated the polycarbonate cage from the wheel attachment, thus preventing access to the wheel. Wheel rotations were recorded daily after each 2-h session. The rats were returned to the same chamber in which they previously self-administered cocaine in the afternoon on day 14 of abstinence to habituate them to their chambers overnight.

#### Reinstatement of cocaine seeking

Following the fourteenth day of abstinence, the rats were tested under an extinction/cue-induced reinstatement protocol using methods previously described.^15^ Briefly, at the beginning of each of the 1-h extinction session, the left lever was extended into the operant chamber, but responses did not have any programmed consequence. The lever retracted at the end of each session. All the animals were given a minimum of six, 1-h extinction sessions separated by 5 min until they reached the extinction criterion of fewer than 15 responses per hour on the previously active lever (generally six sessions, but no more than nine). The cue-induced reinstatement session began 5 min after the last extinction session. At the beginning of this session, a single 5-s presentation of cues formerly associated with cocaine (stimulus light and the sound of the pump) was given. Subsequently, the responses on the previously active lever presented these stimuli under an FR1 schedule.

### Data analysis

All of the self-administration data were objectively measured using Med Associates data collection software, normally distributed and analyzed without the use of condition blinding. The data for cocaine intake over the 10-day extended-access period, wheel running during the first 7 days of access, responding during the six 1-h extinction sessions and responding during the last extinction as compared with the 1-h reinstatement session were analysed by repeated-measures analysis of variance. Total extinction responding during all sessions completed (six to nine sessions), and distance run averaged over the first 7 days and the entire wheel-running period were analyzed using univariate analysis of variance. Similar analyses were also conducted to assess for group differences in body weight before abstinence (as measured at the conclusion of extended-access self-administration period), as well as percent change from this baseline in body weight over abstinence (as measured at the end of the abstinence period and before the reinstatement testing). The association between distance run and total extinction and reinstatement responding was assessed using the Pearson Correlation Coefficient. *Post hoc* comparisons were made using the Fisher's least significant difference controlling for family-wise error. The statistics were run using SPSS 23 (IBM, Armonk, NY, USA) with the alpha set at *P*⩽0.05. All the data are plotted as means±s.e.m.

## Results

### Levels of cocaine self-administration, wheel running and body weight

**Figure 1:**
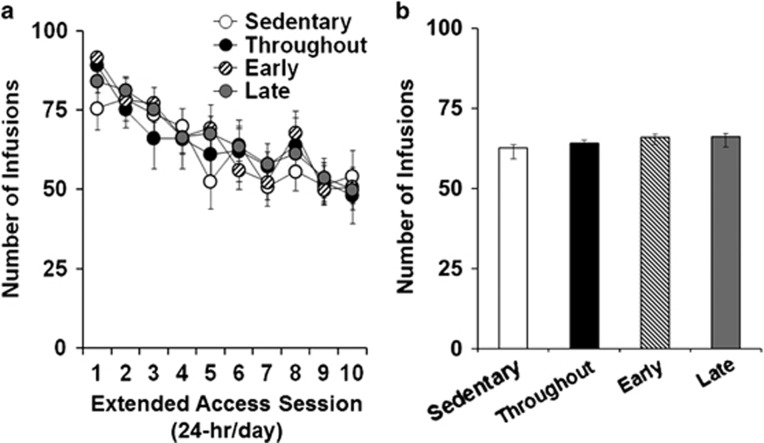
Daily patterns and levels of cocaine self-administration did not vary between the four groups (sedentary, *n*=10; throughout, *n*=10; early, *n*=12; late, *n*=14) during the 10-day extended-access period before abstinence and subsequent exercise/control manipulations. (**a**) Mean (±s.e.m.) number of cocaine infusions self-administered by each of the four groups as a function of extended-access day. (**b**) Mean (±s.e.m.) daily cocaine infusions self-administered by each of the four groups averaged across the 10-day extended-access period.

**Figure 2:**
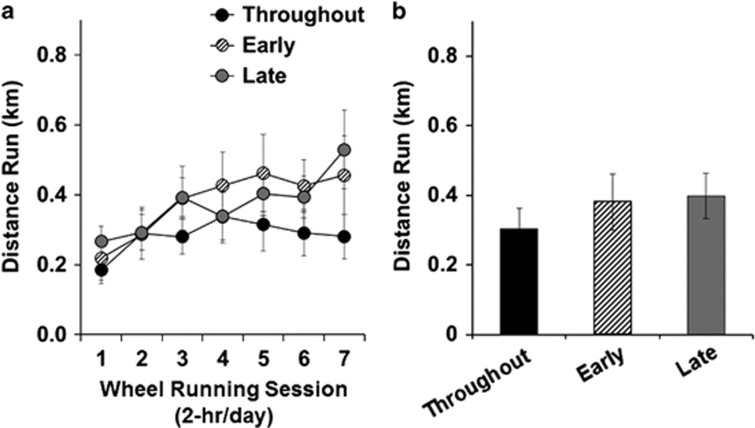
Daily patterns and levels of wheel running did not differ between the three groups given access to an unlocked running wheel (throughout, *n*=10; early, *n*=12; late, *n*=14). (**a**) Mean (±s.e.m.) distance run by each group during the first seven wheel-running sessions. (**b**) Mean (±s.e.m.) daily distance run by each group averaged across all wheel-running sessions.

Before abstinence and the subsequent exercise/control manipulations, each of the groups showed similar patterns (Figure 1a) and levels of cocaine intake (Figure 1b) during the 10 days of extended-access self-administration (*P*-values >0.05). The daily patterns and levels of wheel running during the first 7 days of access to the wheel did not differ statistically between the three groups given access to an unlocked running wheel (*P*>0.05; Figure 2a). The average levels of running during abstinence were also statistically similar between each of these groups both when analyzed as the first 7 days (days 1–7 for early and throughout, and days 8–14 for late) as well as across the entire wheel-access period (Figure 2b; *P*>0.05). The baseline body weight at the end of extended-access self-administration period did not differ between groups (mean±s.e.m.; early: 384±11 g; late: 368±15 g; throughout: 366±19 g; sedentary: 376±14 g; *P*>0.05). In addition, the percent change in body weight from this baseline over abstinence did not differ between the groups (early: 28±5% late: 24±5% throughout: 21±4% sedentary: 26±2%% *P*>0.05). Thus, before abstinence and subsequent exercise/control manipulations, the groups self-administered a similar amount of cocaine, and those given access to an unlocked wheel during abstinence ran at similar levels. The average change in body weight during abstinence also did not differ between the groups.

### Effect of timing of exercise availability on cocaine seeking

**Figure 3:**
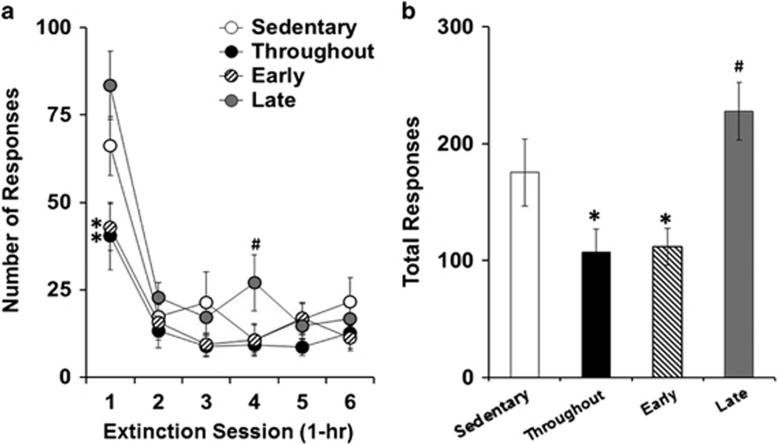
Wheel running beginning during early abstinence decreased levels of subsequent extinction responding (sedentary, *n*=10; throughout, *n*=10; early, *n*=12; late, *n*=14). (**a**) Mean number of responses (±s.e.m.) during each of the 6-h extinction sessions. (**b**) Total responses (±s.e.m.) during each of the extinction sessions completed (six to nine). An asterisk (*) indicates a significant difference from both the sedentary and late groups (*P<0.05). A pound sign (^#^) indicates a trend for a difference from all the other groups (**a**) and from the sedentary group (**b**).

Timing of wheel running during abstinence significantly affected the levels of subsequent extinction responding (group effect, F_3,42_=5.89, *P*<0.01; group by session effect, F_15,210_=2.12, *P*<0.05; Figure 3a) with significantly lower levels observed in the early and throughout groups as compared with both the sedentary (*P*<0.05, *P*<0.01, respectively) and late groups (*P*<0.01, *P*<0.001, respectively). This decrease by wheel running beginning during early abstinence was particularly apparent in the first extinction session (group effect, F_3,42_=5.83, *P*<0.01). *Post hoc* analysis within this session showed that the early and throughout groups responded significantly less than both the sedentary (*P*<0.05, *P*<0.05, respectively) and late groups (*P*<0.01, *P*<0.001, respectively). There were no differences between the early and throughout groups overall or within any of the extinction sessions (*P*-values >0.05). There was a tendency for higher levels of extinction responding in the late group as compared with all the others groups during hour 4; however, the overall group difference within this session did not reach statistical significance (*P*=0.086). An analysis of total levels of extinction responding during each of the sessions completed (six to nine sessions) revealed a significant overall effect of group (F_3,42_=7.21, *P*<0.001; Figure 3b) with results showing that the early and throughout exercise groups had significantly lower levels of extinction responding than both the sedentary (*P*<0.05, *P*<0.05, respectively) and the late groups (*P*<0.001, *P*<0.001, respectively). The late group tended to have higher levels of extinction responding than the sedentary group; however, this effect did not reach statistical significance (*P*=0.099). The levels of total extinction responding did not differ significantly between the early and throughout groups (*P*>0.05).

**Figure 4:**
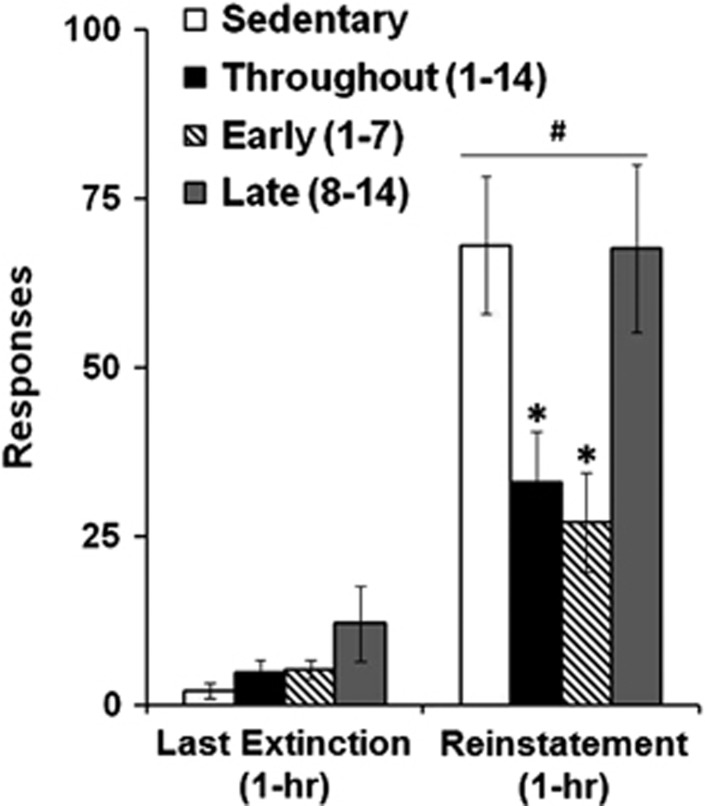
Wheel running beginning during early abstinence decreased levels of subsequent cue-induced reinstatement responding (sedentary, *n*=10; throughout, *n*=10; early, *n*=12; late, *n*=14). Mean number of responses (±s.e.m.) during the last extinction session as compared with the reinstatement session. A bar plus a pound sign (^#^) indicates a significant difference from the last extinction session for all groups. An asterisk (*) indicates a significant difference from both the sedentary and late groups (*P*<0.05).

Timing of exercise also affected cue-induced reinstatement responding and followed the same trends as those seen with extinction responding (group effect, F_3,42_=4.89, *P*<0.01; group by session effect, F_3,42_=3.97, *P*<0.05; Figure 4). Specifically, while all the groups responded at similar levels during the last extinction session (group effect, *P*>0.05), responding increased to a significantly lesser extent during reinstatement for the groups given access to an unlocked running wheel during early and throughout abstinence (group effect, F_3,42_=4.83, *P*<0.01) as compared with both the sedentary (*P*<0.01, *P*<0.05, respectively) and the late exercise (*P*<0.01, *P*<0.01, respectively) groups. The levels of reinstatement responding did not differ between the early and throughout groups (*P*>0.05) or between the late and sedentary groups (*P*>0.05). Thus, following extended-access cocaine self-administration, exercise during early and throughout abstinence, but not during late abstinence, significantly attenuated both extinction and cue-induced reinstatement responding. Exercise during late abstinence, as compared with sedentary conditions, tended to increase some measures of cocaine seeking, specifically hour 4 of extinction responding and total extinction responding.

### Relationship between levels of running and subsequent cocaine seeking

An analysis of the relationship between levels of running and total extinction and reinstatement responding revealed significant correlations for the throughout group. In this group, the levels of running accounted for approximately 85% of total extinction responding (*r*=0.94, *P*<0.01), and 34% of reinstatement responding (*r*=0.58, *P*<0.05). Surprisingly, the data from the early and late groups showed no significant relationships between the levels of running and either measure of subsequent cocaine seeking (*P*>0.05, Table 1).

## Discussion

A number of clinical and preclinical studies have demonstrated the potential for exercise as an intervention for drug addiction.^14^ Few studies, however, have explored the exercise conditions that best prevent drug seeking and relapse. On the basis of a neurobiological framework, we predicted that the timing of exercise availability during abstinence may be critical for producing an efficacious response. Consistent with our hypothesis, we showed that exercise beginning during early abstinence robustly attenuated subsequent cocaine seeking as compared with sedentary conditions. Notably, the levels of extinction and reinstatement responding observed in animals who exercised during early abstinence were comparable to those observed in animals who exercised throughout abstinence. This finding is striking given that the time from the last exercise session to extinction/reinstatement testing was much longer in the early exercise group. In contrast, exercise during later abstinence was not effective and levels of cocaine seeking in this group were equal to, and in some cases tended to be higher, than those observed under sedentary conditions. These protective effects appear to be due specifically to exercise and not environmental enrichment given that all groups were exposed to a wheel (locked or unlocked). These findings may have strong translational value in that they suggest that exercise needs to be introduced early in the course of treatment for it to be effective. This point may be critical because several exercise trials are being conducted for addiction treatment in humans, but most introduce exercise well after the initiation of abstinence.^42^

Our findings showing that exercise beginning during early abstinence effectively reduces cocaine seeking are consistent with our previous work and recent work by others. For example, several studies have demonstrated an acute beneficial effect of exercise at reducing cocaine seeking when exercise immediately precedes or is concurrent with reinstatement testing.^43^ Zlebnik and Carroll^44^ also showed that wheel-running exercise during abstinence and immediately before extinction/reinstatement testing attenuated the incubation of cocaine seeking over abstinence. Our work, as well as recent work by others, has shown that the beneficial effects of exercise also extend beyond an acute response with results showing a reduction in cocaine seeking even when testing and exercise are not contemporaneous.^15, 16, 17, 18^ Our current findings provide strong further support for a persistent beneficial effect of exercise with results showing that exercise beginning during early abstinence robustly attenuated cocaine seeking, even when a full week separated the last exercise session from the extinction/reinstatement test. In fact, despite a significantly longer time between exercise and subsequent extinction/reinstatement testing, early exercise produced a similar decrease in subsequent cocaine seeking as compared with throughout exercise. These findings indicate a persistent beneficial effect of exercise and suggest that the timing of exercise availability is even more critical than length of exposure. Although these findings in males are striking and relevant to current clinical trials, future research is needed to determine whether such effects extend to females.

This idea that the timing of exercise availability is critical for producing an efficacious response is further supported by our findings showing that, in contrast to exercise during early and throughout abstinence, exercise during late abstinence was not effective. In fact, in some cases, exercise during late abstinence tended to increase cocaine seeking. These tendencies for an increase rather than a decrease were predicted by our neurobiological framework^14^ and are consistent with other studies showing that certain exercise conditions increase rather than decrease addiction-related behaviors. For example, a history of chronic unlimited exercise on a wheel has been reported to increase cocaine-induced place preference when exercise occurred before cocaine conditioning.^23, 45^ Chronic forced running on a treadmill has also been shown to enhance rather than attenuate subsequent cocaine-primed reinstatement.^24^ Our current findings show that even a modest level of voluntary exercise (2 h per day) beginning during late abstinence has the potential to increase cocaine seeking and relapse vulnerability.

These differential effects of late versus early exercise were also predicted by our neurobiological framework.^14^ Specifically, protracted abstinence results in compensatory increases in several mesolimbic signaling pathways, and exercise is known to increase some of these same pathways.^28, 29, 33, 34, 36^ As such, we predicted that when exercise was available during late abstinence it could result in the amplification of the pathways underlying the incubation of cocaine craving. Conversely, during early abstinence when signaling at some of these pathways is depressed, the increase caused by exercise has the potential to block the development of later compensatory changes. Although the exact pathways involved in mediating these effects are not yet known, they likely include dopamine and BDNF signaling. During early abstinence, the activity of both dopamine and BDNF is decreased.^33^ The reduction in dopamine activity has been linked to withdrawal symptoms,^46^ and BDNF is one of the few markers that positively associates with the incubation of cocaine seeking over abstinence.^29, 32, 33, 47, 48^ Wheel running has the ability to upregulate both dopamine and BDNF under normal conditions,^35, 36^ and recent studies in both humans and animals have shown that exercise can counteract the reduction in dopamine receptors caused by methamphetamine abuse.^49, 50^ Thus, wheel running may induce time-dependent effects on cocaine seeking through its ability to modulate dopamine and BDNF signaling. Wheel running also elevates BDNF signaling through long-term epigenetic mechanisms,^51^ and these types of changes may explain the persistent effects that we observed here following exercise during early abstinence. In support of this idea, our recent findings show that wheel running throughout abstinence reduced BDNF gene expression in males as well as cocaine-seeking behavior.^16^ Although these findings provide strong support for a potential role of dopamine and BDNF signaling as mechanisms underlying the efficacy of exercise, further research is needed to confirm their involvement and to examine other potential mechanisms.

Our results also show that the amount of exercise during abstinence predicted subsequent cocaine seeking, but only following exercise throughout abstinence. Specifically, we found that although exercise throughout abstinence robustly attenuated subsequent cocaine seeking, individuals that ran at high levels also exhibited higher levels of cocaine seeking. Although this finding is surprising given the results demonstrating the dose-dependent effectiveness of exercise at reducing cocaine seeking, it is consistent with several previous studies indicating a predictive relationship between exercise and addiction vulnerability. Specifically, levels of activity, as measured by locomotor behavior or by levels of wheel running predict vulnerability during all the phases of the addiction process, including reinstatement.^52, 53^ Therefore, while wheel running throughout abstinence consistently attenuated relapse vulnerability, high levels of running resulted in a slightly smaller decrease due to an innate vulnerability in those animals. Notably, no such relationship was observed following exercise during only early or late abstinence suggesting that individual differences predictive of vulnerability are obscured under these conditions. For example, it is possible that when exercise is timed properly, for example, available during early abstinence, even a little bit of exercise could effectively reduce the signaling deficits inherent to early abstinence and block the compensatory changes that lead to enhanced cocaine seeking during later abstinence. Conversely, exercise beginning during later abstinence, when mesolimbic pathways are sensitized, may result in minimal changes due to a ceiling effect either at a behavioral or neurobiological level. Although these correlational data present an intriguing trend, further research is needed to fully understand their mechanisms.

One limitation to our study is that vulnerability to relapse was assessed after only 2 weeks of abstinence. Another limitation is that the length of exercise treatment (1–2 weeks) that we used in our animal model is shorter than those typically used in human studies (for example, 12 weeks).^10^ Future work should investigate the persistence of the effects of early exercise at later time points during abstinence. Despite these differences, we believe that our model is a strong reflection of the potential efficacy of early exercise interventions in human cocaine abusers.

In conclusion, this study demonstrates that the timing of exercise availability is critical for reducing cocaine seeking. These findings may have important implications for exercise programs used to facilitate addiction recovery. Although implementing exercise-based programs in humans are not without their challenges, our results suggest that even a little bit of exercise properly timed during early abstinence can effectively decrease subsequent drug seeking and relapse vulnerability. This is important because drug abusers may be reluctant to exercise regularly, particularly during the early days of recovery while they are experiencing drug withdrawal symptoms. However, exercise programs beginning during withdrawal appear to be possible in humans, with recent results showing that a majority of drug abusers are both willing to exercise during early abstinence^54^ and are able to complete an exercise-based treatment program.^13, 55, 56^ Taken together, these findings indicate that exercise programs initiated during early cocaine abstinence will likely produce a maximally efficacious response during addiction recovery.

## Figures and Tables

**Table 1:** Correlations (*r*) between distance run averaged across each of the wheel-running sessions and total extinction and reinstatement responding

	*Total extinction*	*Reinstatement*
Early	−0.12	−0.28
Late	−0.06	0.09
Throughout	0.94*	0.58*

*P<0.05.
